# Hypervirulent and hypermucoviscous extended-spectrum β-lactamase-producing *Klebsiella pneumoniae* and *Klebsiella variicola* in Chile

**DOI:** 10.1080/21505594.2020.1859274

**Published:** 2020-12-29

**Authors:** F. Morales-León, A. Opazo-Capurro, C. Caro, N. Lincopan, A. Cardenas-Arias, F. Esposito, V. Illesca, M. L. Rioseco, M. Domínguez-Yévenes, C. A. Lima, H. Bello-Toledo, Gerardo González-Rocha

**Affiliations:** a Laboratorio de Investigación en Agentes Antibacterianos, Facultad de Ciencias Biológicas, Universidad de Concepción, Concepción, Chile; b Millennium Nucleus for Collaborative Research on Bacterial Resistance, Chile; c Departamento de Farmacia, Universidad de Concepción, Concepción, Chile; d Department of Microbiology, Institute of Biomedical Sciences, Universidade de São Paulo, São Paulo, Brazil; e Department of Clinical Analysis, School of Pharmacy, Universidade de São Paulo, São Paulo, Brazil; f Unidad de Microbiología, Hospital Dr. Hernan Henriquez Aravena, Temuco, Chile; g Laboratorio de Microbiologia, Hospital de Puerto Montt, Puerto Montt, Chile

**Keywords:** *Klebsiella pneumoniae* complex, virulence, hypermucoviscous, ESBL, multidrug-resistance

## Abstract

Convergence of virulence and antibiotic-resistance has been reported in *Klebsiella pneumoniae*, but not in *Klebsiella variicola*. We, hereby, report the detection and genomic characterization of hypervirulent and hypermucoviscous *K. pneumoniae* and *K.variicola* recovered in Chile from health-care associated infections, which displayed resistance to broad-spectrum cephalosporins. One hundred forty-six *K. pneumoniae* complex isolates were screened by hypermucoviscosity by the “string test.” Two hypermucoid isolates, one hypermucoviscous *K. pneumoniae* (hmKp) and one *K. variicola* (hmKv), were further investigated by whole-genome sequencing. *In vivo* virulence was analyzed by the *Galleria mellonella* killing assay. *In silico* analysis of hmKp UCO-494 and hmKv UCO-495 revealed the presence of multiple antibiotic-resistance genes, such as *bla*_CTX-M-1_, *bla*_DHA-1_ and *bla*_LEN-25_ among others clinically relevant resistance determinants, including mutations in a two-component regulatory system related to colistin resistance. These genetic features confer a multidrug-resistant (MDR) phenotype in both strains. Moreover, virulome *in silico* analysis confirmed the presence of the aerobactin gene *iutA*, in addition to yersiniabactin and/or colicin V encoding genes, which are normally associated to high virulence in humans. Furthermore, both isolates were able to kill *G. mellonella* and displayed higher virulence in comparison with the control strain. In summary, the convergence of virulence and the MDR-phenotype in *K. pneumoniae* complex members is reported for the first time in Chile, denoting a clinical problem that deserves special attention and continuous surveillance in South America.

## Introduction

*Klebsiella pneumoniae* complex includes *K. pneumoniae* sensu stricto, *K. quasipneumoniae* subsp. *quasipneumoniae, K. quasipneumoniae* subsp. *similipneumoniae, K. variicola* subsp. *variicola, K. variicola* subsp. *tropica, K. quasivariicola*, and *K. africana*, respectively [1]. Among members of this complex, *K. pneumoniae* and *K. variicola* have been widely recognized as important opportunistic human pathogens commonly involved in hospital-acquired infections (HAIs) [2,3]. The clinical importance of these species has been associated with multidrug-resistance, mediated by the expression of extended-spectrum β-lactamases (ESBLs) and carbapenemases [4,5], and more recently with colistin resistance [6,7]. Lately, convergence of virulence and antibiotic-resistance has been reported in *K. pneumoniae* [8]. In this regard, hypervirulent *K. pneumoniae* (hvKp) isolates have been defined under the following criteria: i) occurrence of the hypermucoviscous (hmKp) phenotype, as determined by a positive “string test”; ii) presence of the *rmpA* gene, which regulates the capsule biosynthesis; and iii) presence of the aerobactin genes *iucA/iutA* [9,10]. Similarly to *K. pneumoniae, K. variicola* can also display the hypermucoviscous (hmKv) and/or hypervirulent (hvKv) phenotypes [1]. Currently, hvKp isolates have been reported mainly in Asia, Europe and North America, and more recently in South America [9], where sporadic reports have been restricted to Argentina and Brazil [10–12]. Hence, the aim of our study was to detect and characterize hypervirulent and hypermucoviscous ESBL-producing *K. pneumoniae* and *K. variicola* isolates recovered from Chilean hospitals.

## Materials and methods

### K. pneumoniae complex isolates and antibiotic susceptibility testing

One hundred forty-six non-repetitive *K. pneumoniae* complex isolates collected between 2011 and 2018 in Chile, were investigated. All isolates were recovered from nosocomial infections and were initially identified by each hospital laboratory as third-generation cephalosporin-resistant *K. pneumoniae*. Species identification was confirmed by conventional PCR according to previously described [13]. Antibiotic susceptibility testing to imipenem, ertapenem, meropenem, ceftriaxone, cefpodoxime, cefotaxime, ceftazidime, amoxicillin/clavulanic acid, amikacin, tobramycin, kanamycin, gentamicin, ciprofloxacin, levofloxacin and tetracycline was performed by the Kirby-Bauer method. ESBL-production and colistin susceptibility were determined by the combined disc test and the broth microdilution method, respectively [14].

### Phenotypic identification of hypermucoviscous isolates

The hypermucoviscous phenotype was determined by the “string test” [15]. In brief, when a bacteriological loop was able to generate a viscous filament ≥5 mm in length by stretching bacterial colonies growth at 37ºC by 18–24 h on a blood agar plate, the isolate was considered as positive, thus defined as hypermucoviscous. Two isolates resulted positive for the “string test,” therefore, subsequent experiments included both strains.

### Whole-genome sequencing (WGS) and in silico analyses of hypermucoviscous isolates

Total DNA of both hypermucoviscous isolates was extracted for whole-genome sequencing (WGS) using the Wizard® Genomic DNA Purification kit (Promega, USA) following the manufacturer’s protocol. Sequencing was performed by the Illumina MiSeq platform (2 × 250 bp paired end reads) with libraries prepared by the NexteraXT kit (Illumina), with a coverage of 30x.

*De novo* assembly was carried out by using the SPAdes software, version 3.9 (https://cge.cbs.dtu.dk/services/SPAdes/) with default values. Later, the assembled genomes were used to screen for genes for antibiotic-resistance, plasmids and virulence using the ResFinder v3.2, PlasmidFinder v2.1 and Virulence Finder v2.0 tools available at the Center for Genomic Epidemiology server (https://cge.cbs.dtu.dk/services/). Resistome (antibiotics, heavy metals, and disinfectants) was further predicted by the comprehensive antibiotic resistance database (CARD) (https://card.mcmaster.ca/), and ABRicate v0.9.8 (https://github.com/tseemann/abricate) using the BacMet2 database (http://bacmet.biomedicine.gu.se), respectively, considering a ≥ 90% similarity criteria. Genome annotation was accomplished using the NCBI Prokaryotic Genome Annotation Pipeline (PGAP) web-service (http://www.ncbi.nlm.nih.gov/genome/annotation_prok). Sequence types (STs) were determined for *K. pneumoniae* and *K. variicola* through the bioinformatic tools available at https://cge.cbs.dtu.dk/services/MLST/andhttp://mlstkv.insp.mx, respectively. Capsular serotypes (K-locus) and phylogenetic analysis of the *ybt* locus were predicted by Kleborate (https://github.com/katholt/Kleborate). Mutations in chromosomal genes *mgrB, phoPQ,* and *pmrAB* were analyzed with local BLAST+ DB using *K. pneumoniae* MGH78578 or *K. variicola* DSM 15,968 (accession number NC_009648.1 and NZ_CP010523.2) genomes as colistin-susceptible references. In order to predict the functional effect of amino acid substitutions, we used the PROVEAN web server (http://provean.jcvi.org/index.php).

We studied mutations in *wzc, rcsAB,* and *lon* genes in UCO-494 utilizing the *K. pneumoniae* (accession numbers LT174540 and JCMB01, respectively) genome as reference [16]. For all mutation, bioinformatic analysis was performed using the UGENE 1.32.0 Software.

Both UCO-494 and UCO-495 genomes have been deposited at DDBJ/ENA/GenBank under the accession numbers VSSY00000000.1 and VSSZ00000000.1, respectively.

### Serum bactericidal assay and virulence behavior in the Galleria mellonella infection model

Serum bactericidal activity was analyzed according to previously described [17], with minor modifications. Briefly, 250 μL of a bacterial inoculum of 5 × 10^6^ CFU/ml were mixed with 750 μL of fresh human serum. Then, viable bacterial cell count was performed in tryptone soy agar (TSA) plates. A *K. pneumoniae* isolate that was previously characterized as hypervirulent in our laboratory was used as positive control, while serum inactivated at 56°C for 30 min was utilized as blank. All experiments were performed in triplicate. A bacterial survival of <1% after 3 h of incubation with serum was considered as susceptible. On the other hand, survival percentages of 1–90% or >90% were considered as intermediate and resistant, respectively [18]. Additionally, in order to compare the levels of virulence of hmKp UCO-494 and hmKv UCO-495, the *G. mellonella* infection model was utilized [19]. *K. quasipneumoniae* subsp. *similipneumoniae* ATCC700,603 and hvKp k1/ST23 UCO-448 [10] were used as negative and positive hypervirulent controls, respectively. Larvae survival was analyzed during 96 h, and Kaplan-Meier killing curves of *G. mellonella* were generated using the log rank test with *p* < 0.05. Each assay was performed in triplicate.

### Capsular-polysaccharide (CPS) quantification and estimation of capsular size

Total capsular-polysaccharide (CPS) of hmKp UCO-494 and hmKv UCO-495 was estimated according to the phenol-sulfuric acid method, after extraction using zwittergent 3–14 [20], and incubated in tryptone soy broth (TSB) at 37ºC for 18 h with agitation. The estimation of capsular size was carried out by transmission electron microscopy (TEM) of a bacterial inoculum incubated at 37ºC for 24 h [21]. Prior to microscopy, the samples were centrifuged at 3,000 rpm for 5 min and washed once with PBS buffer.

### Biofilm assay

Biofilms-quantification was performed as previously described [22]. In brief, a colony from each strain was grown overnight in TSB at 37°C. From this culture, 10 μL of a bacterial suspension was used to inoculate 96-well polystyrene plates containing 90 μL of TSB, and these plates were incubated at 37°C for 24 h. Subsequently, the medium was removed from the plates and each wells was washed three times with water. Immediately, the samples were stained with 125 μL of 0.1% crystal violet for 15 min. Excess dye was removed by rinse 4 times in water, and dried during 10 min at 65°C. Afterward, 125 μL of acetic acid solution (30% v/v) were added and then incubated for 15 min at room temperature. Then, 125 μL of the solubilized crystal violet were transferred to a new 96-well polystyrene plates and color intensity was determined at a 550 nm using a spectrophotometer. *K. pneumoniae* ATCC 700603 strain was used as positive control, and acetic acid solution (30% v/v) was used as a negative control. Biofilm-formation abilities were defined as follows: i) absorbance values between 0.084 and 0.168 (2x – 4x blank absorbance) were considered as low biofilm-forming strains; ii) values ranging between 0.168 and 0.252 (4x – 6x blank absorbance) were considered as medium biofilm-forming strains, whereas iii) strains displayed absorbance values higher than 0.252 (>6x blank absorbance) were classified as high biofilm formers [23].

## Results

Two hypermucoviscous isolates exhibiting a positive string test were identified as *K. pneumoniae* (UCO-494) and *K. variicola* (UCO-495) (Table 1). UCO-494 and UCO-495, belonging to the ST1161 and ST173 lineages, respectively, were isolated from blood and catheter cultures of ICU patients, admitted at two different hospitals located in southern Chile (Table 1). Both isolates were resistant to aminoglycosides and broad-spectrum cephalosporins. UCO-494 was additionally resistant to ertapenem, levofloxacin and ciprofloxacin, remaining susceptible to imipenem, meropenem, and tetracycline. Additionally, colistin-resistance in UCO-494 and UCO-495 was associated with MIC values of 8 and 16 μg/mL, respectively (Table 1). Resistome analysis revealed the presence of the ESBLs and cephalosporinases encoding genes *bla*_CTX-M-1_, *bla*_SHV-187_ and *bla*_DHA-1_ in *K. pneumoniae* UCO-494 and *bla*_SHV-12_ and the *bla*_LEN-25_ genes in *K. variicola* UCO-495 (Table 1). Moreover, ertapenem resistance in *K. pneumoniae* UCO-494 was associated with a deletion in the *ompK35* gene, leading to porin deficiency, and also linked to the presence of the *ompK37* gene, which has been associated with reduced permeability to carbapenems [24,25]. Additionally, *K. pneumoniae* UCO-494 harbored the *aac(6ʹ)-Ib; aac(6ʹ)-Ib-cr, aadA1* and *aadA2* and *K. variicola* UCO-495, the *aph(3”)-Ia, aph(6)-Id* and *aph(3”)-Ib* aminoglycosides resistance genes (Table 1).

**Table 1.** Strain characteristic, MLST, capsular locus type, resistome and virulome of UCO-494 and UCO-495

Phenotype	UCO-494 hmKp	UCO-495 hmKv
*Origin*	Blood	Catheter
*year*	2012	2012
*String test*	+	+
*ST*	1161	173
K-locus*	KL19	KL25
*ybt*	ybt14 ICEKp5	-
*ybST*	327–1LV	-
*O-locus*	O1v2	-
*ESBL combined disc test*	+	+
**WGS data**		
*Contig number*	462	226
*Genome size (bp)*	6,400,426	5,982,509
*GC%*	56,4%	56,1%
*CDS; pseudogenes; tRNA*	6668;203;95	5968;169;79
*Resistance profile*	ERT, CIP, LEV AMK, KAN, GEN, TOB, AMP, CTX, CAZ, AMC	TET, STX, W, CPD, CRO, AMK, KAN, GEN, TOB, AMP, CTX, CAZ, FEP, AMC
*MIC colistin*	8 μg/mL	16 μg/mL
**Resistome**		
*Antibiotic resistance genes*	*sul1; sul2; arr-2; dfrA12; aadA1; aadA2; aac(6`)-Ib; aac(6`)-Ib-cr; oqxA/B; qnrB19; qnrB4; bla_CTX-M-1_; bla_DHA-1_; bla_OXA-10_; bla_OXA-9_; bla_SHV-187_; gyrA83L; gyrA87Y; parC80I*	*bla_LEN-25_; bla_SHV-12_; bla_TEM-1B_; oqxA; oqxB; aph(3”)-Ia; aph(6)-Id; aph(3”)-Ib; tet(D)*
*colistin mutation gen*	*PmrB: Gly256Arg (G766C)*	*PmrA: Thr146Ala (A436G); PmrB: Ser170Ala (G508T); PhoQ: Asp152Glu (T456G)*
*Heavy-metal resistance genes*	arsenic *(arsCDBAH*); magnesium/cobalt/nickel/manganese *(corA*); glyphosate (*phnMLKJI*); quaternary ammonium (*emrD – qacE∆1*)	arsenic (*arsBCRD*), cobalt/manganese (*corC*), cobalt/magnesium (*mgtA*), magnesium/cobalt/nickel/manganese (*corA*): tellurium resistance gen (*terW* and *terZCD)*
**Virulome**		
*Virulence genes*	Enterobactin (*entB; entF; ycfH; entD), urea(ureA), alantoin (allS), aerobactin (iutA), fimbria type 1 (fimABCDFEGH), fimbria type 3 (mrkABCDF), yersiniabactin (irp1; irp2; fyuA; ybtAES), colicin V (cvpA; cvaA), biofilm (treC; sugE), ECP (ecpABCDE)*	*Urea (ureA), alantoin (allS), aerobactin (iutA), fimbria type 1 (fimABCDFEGH), fimbria type 3 (mrkABCDF), colicin V (cvpA; cvaA), biofilm (treC; sugE), ECP (ecpABCDE), KFU (kfuABC)*
*Plasmids*	ColRNAI; IncA/C2; IncFIB (3); IncFII	IncFIB; IncFII; IncHI2; IncHI2A

hmKP, hypermucoviscous *Klebsiella pneumoniae*; hvKP, hypervirulent *Klebsiella pneumoniae*; *Capsular polisacharide concentration in OD_650nm_ 2.0. Significative difference with p-value equal to 0.0001 in *t* test. ERT, ertapenem; CIP, ciprofloxacin; LEV, levofloxacin; AMK, amikacin; KAN, kanamycin; GEN, gentamicin; TOB, tobramycin; AMP, ampicillin; CTX, cefotaxime; CAZ, ceftazidime; FEP, cefepime; ; CPD, cefpodoxime; CRO, ceftriaxone; AMC, amoxicillin-clavulanic acid; W, trimethoprim; TET, tetracycline; SXT, sulphamethoxazole-trimethoprim .

Importantly, both isolates were resistant to colistin (Table 1). From WGS data, we predicted in *K. pneumoniae* UCO-494 (colistin_MIC_ 8 ug/mL) a Gly256Arg (G766C) amino acid substitution in PmrB, while in *K. variicola* UCO-495 (colistin_MIC_ 16 ug/mL) we predicted a Ser170Ala (G508T) amino acid substitution in PmrB, Thr146Ala (A436G) in PmrA and Asp152Glu (T456G) in PhoQ. All amino acid substitutions were neutral by PROVEAN.

Fluoroquinolone resistance in *K. pneumoniae* UCO-494 strain was mediated by *aac(6ʹ)-Ib-cr, oqxA, oqxB, qnrB19* and *qnrB4* genes and *gyrA* (83 L, 87Y) and *parC* (80I) mutations. Moreover, *K. pneumoniae* UCO-494 strain harbored the ColRNAI, IncA/C2, IncFIB and IncFII plasmids. On the other hand, *K. variicola* UCO-495 was susceptible to fluoroquinolones and additionally carried IncF-like plasmids (Table 1).

Furthermore, in *K. variicola* UCO-495, we found diverse metal-resistance systems, such as the arsenic (*arsBCRD*), cobalt/manganese (*corC*), cobalt/magnesium (*mgtA*), magnesium/cobalt/nickel/manganese (*corA*) and tellurium resistance genes t*erW* and *terZCD*. Moreover, *K. pneumoniae* UCO-494 contained the arsenic *(arsCDBAH)* and magnesium/cobalt/nickel/manganese *(corA)* systems. Likewise, were identified the presence of resistance genes to glyphosate (*phnMLKJI*) and quaternary ammonium compounds (*emrD – qacE∆1*) in *K. pneumoniae* UCO-494.

In *K. pneumoniae* UCO-494, phylogenetic analysis of the *ybt* locus revealed 14 lineages (*ybt* locus sequence type YbST 327–1LV) with ICE*Kp5* element, were K-locus KL19 and O-locus O1v2, were also identified. On the other hand, we designated a new ST to MLST *K. variicola*, which corresponded to ST173 (allelic profile *leuS*10; *pgi* 9; *pgk* 6; *phoE* 1; *pyrG* 11; *rpoB* 1; f*usA* 2), whereas *K. pneumoniae* UCO-494 belonged to ST1161 (Table 1).

Virulome analysis of hvKv UCO-495 revealed the presence of the ferric uptake system kfuABC, which has been associated to hypervirulent *Klebsiella* strains [15]. Both isolates contained the aerobactin gene *iutA*, mannose-sensitive type 1 fimbriae *(fimABCD* operon), the mannose-resistant *Klebsiella*-like (type III) fimbriae cluster *(mrkABCDFHIJ*), and the *E. coli* common pilus operon (*ecpABCDE*) and biofilm related (*treC, sugE*) genes, which are associated with mucoviscosity and CPS production [26]. Only hmKp UCO-494 carried additionally the enterobactin (*entB, entF,* and *ycfH*), yersiniabactin siderophore cluster *ybtAEPQSTUX* and the siderophore genes *irp1* and *irp2*, which are considered as genetic markers for high-pathogenicity island [27] (Table 1). It is important to highlight that in both strains the presence of *rmpA/A2* was not identified.

**Figure 1.**
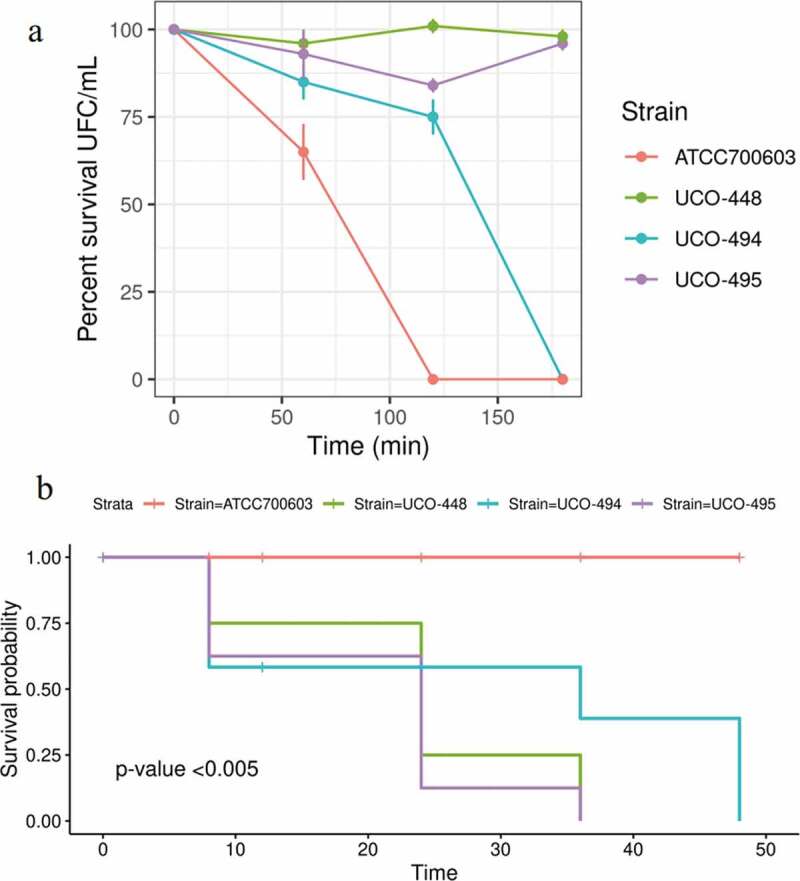
A) Serum bactericidal activity. *K.quasipneumoniae* subsp. *similipneumoniae* ATCC 700603 as negative control; *K. pneumoniae* hypervirulent UC-448 as positive control. b) *K. pneumoniae* UCO-494 and *K. variicola* UCO-495; Kaplan-Meier killing curves of *G. mellonella* larvae; ATCC 700603 as negative control; *K. pneumoniae* hypervirulent UC-448 as positive control; The assay was made with blank, inoculated the larvae with NaCl 0.9%. Data no showed

**Figure 2.**
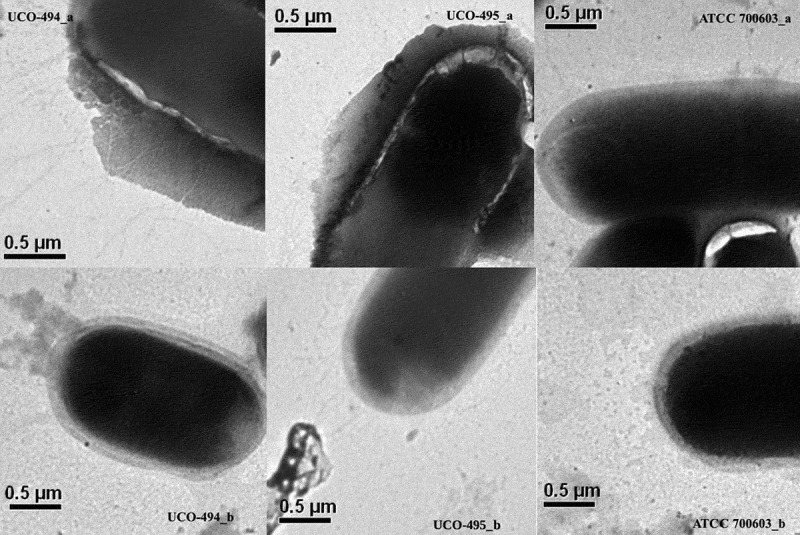
Representative transmission electronic microscopy images of exopolysaccharide capsular UCO-494_a (*K. pneumoniae* UCO-494); UCO-495_a (*K. variicola* UCO-495) and ATCC700603_a (*K.quasipneumoniae* negative control) without washes; UCO494_b (*K. pneumoniae* UCO-494); UCO-495_b (*K. variicola* UCO-495) and ATCC700603_b (negative control) after washes. We estimated of capsular size in 0.124 ± 0.017; 0.097 ± 0.019 µm and 0.091 ± 0.012 µm for UCO-494_b; UCO-495_b and ATCC700603_b, respectively

Interestingly, hmKv UCO-495 was resistant to the bactericidal activity of human serum, while hmKp UCO-494 was susceptible, with 1% survival after 1 h interaction (Figure 1). Curiously, *K. pneumoniae* UCO-494 produced more CPS (155.44 ± 3.68 μg/mL) than *K. variicola* UCO-495 (30.26 ± 0.11 μg/mL). Likewise, UCO-494 displayed a capsular thickness of 0.124 ± 0.017 µm, whereas capsule thickness of UCO-495 was 0.097 ± 0.019 µm (Figure 2). Interestingly, in UCO-494 we predicted a F573S (T1718C) and R608T (G1823C) amino acid substitutions in *wcz* (deleterious by PROVEAN). Moreover, S35N (G104N) amino acid substitution in *crsA* in addition to E142Q (G424C) and R517C (T843C) in *lon* gen was identified. All of these genes were related with hypercapsule production [16].

On the other hand, *K. variicola* UCO-495 killed >75% *G. mellonella* larvae at 24 h post-infection, while *K. pneumoniae* UCO-494 killed 50% of the larvae at 24 h post-infection. Moreover, 100% mortality was observed at 36 and 48 h, respectively (Figure 1). Finally, hmKp UCO-494 displayed a low biofilm-forming ability, since it showed an OD550 nm 0.130 ± 0.003, whereas hmKv UCO-495 was classified as medium biofilm-producer since it displayed an OD550 nm value of 0.246 ± 0.021 [23].

## Discussion

Traditionally, *K. variicola* has been considered as susceptible to most antibiotic classes, but this description has change over time, due to an increase in the MDR-*K. variicola* reports [1]. In South America, there is a single report in Colombia describing a KPC-2-producing *K. variicola* strain, which was resistant to all β-lactams [5].

Worryingly, it is the emergence of hypervirulent-MDR phenotype, especially in *K. variicola* isolated. In this regard, Farzana R *et al.* describe a fatal MDR-hvKv outbreak in neonates in Bangladesh. The isolates contained a *bla*_CTM-M-15_ and *bla*_NDM-1_ genes, among others, in addition to several virulence genes like siderophore (*kfuABC*) and Enterobactin (*entABCDEFHIJ*) associated with hypervirulent phenotype [6]. On the other hand, Lu *et al.* described the first hvKv isolated from blood from a patient with cholangitis in China, which was resistant to colistin (MIC = 8 ug/mL) [28]. These are concordant with our study since we identified an MDR *K. variicola* that was resistant to colistin. In the case of *K. pneumoniae*, colistin-resistant hvKp isolates has been reported previously. Specifically, Lu *et al.* reported five colistin-resistant hmKp strains recovered from blood samples in China [29]. Similar to our findings, these isolates were colistin-resistant and carbapenems-susceptible. Moreover, Huang *et al.* characterized diverse colistin-resistant hmKp isolates that were also resistant to carbapenems, since they produced the KPC-2 carbapenemase [30].

Our findings described the convergent hypervirulent phenotype and colistin-resistance in *K. pneumoniae* and *K. variicola* MDR strains. In this sense, the mutations in genes involved in colistin-resistance might be mediating this phenotype. As described previously, point mutations or deletions in *pmrA* or *pmrB* genes result in the addition of phosphoethanolamine to the lipid A [31]. Moreover, it has been demonstrated *in vivo* the role of PmrAB system, in which it has been associated to intra-macrophage survival and virulence in *K. pneumoniae* [32]. In case of hvKp UCO-494, we identified a point mutation in *pmrB*, similarly to the description of Lagerbäck *et al.*, where a NDM-1-producing *K. pneumoniae* isolate presented an amino acid substitution in G256R in the *pmrB* gen [33], which was related with colistin-resistance *K. pneumoniae* [34]. Furthermore, it is important to highlight that the mechanism of colistin-resistance in hvKv UCO-495 was mediated by chromosomal mutations in the two-component system PhoPQ, especially in the D150G substitution in PhoP. Even though mutations in these systems are associated to colistin-resistance [30], general data of molecular mechanisms of colistin-resistance in *K. variicola* are scarce; therefore, our results describe a non-classical *pmrAB* and *phoQ* mutations in this species [7]. In this regard, we determined that these mutations are neutral according to *in silico* models, in consequence, *in vivo* studies should be performed in order to determine if they have an impact on colistin-resistance [16,35].

WGS analyses reflect a widely diverse resistome. In this sense, the *bla*_LEN-25_ gene was detected in the *K. variicola* UCO-495 genome, which corresponds to an intrinsic-chromosomal β-lactamase. Furthermore, we found that hvKv UCO-495 strain was resistant to cephalosporins, which might be mediated by *bla*_SHV-12,_ while hvKp UCO-494 resistance was mediated by *bla*_CTX-M-1._ In this case, there are some reports of convergence of hypervirulent phenotype and ESBL genes in *K. pneumoniae*. For instance, hypervirulent and ESBL-producing have been linked to several ESBLs genes, such as *bla*_CTX-M-14_, *bla*_CTX-M-18_, *bla*_CTX-M-3_ and *bla*_SHV-12_ [36,37,38].

In case of heavy-metal resistance genes, we found in hvKv UCO-495, the tellurium resistance genes *terW* and *terZCD*, which are related to the plasmid pKV8917 [39] in hvKp and hvKv strain [1,40]. These genes were not detected in hvKp UCO-494. Relevantly, we identified the presence of the quaternary-ammonium resistance gene *emrD* in *K. pneumoniae* UCO-494. As note, these compounds have been heavily used during the SARS-CoV-2 pandemic as disinfectants, which could have an important ecological impact on selecting MDR-bacterial isolates due to selective pressure [41].

Furthermore, Moura *et al.* identified a *K. pneumoniae* serotype K19 isolate in Brazil [10]. In this study, the authors determined that this serotype has a similar killing ability compared to hypervirulent K1-isolates [10]. Moreover, the Brazilian isolate produced the ESBL CTX-M-15, which belongs to the same group of the ESBL detected in hvKp UCO-494 isolate (CTX-M-1) [42]. These findings suggest that this serotype could be endemic to South America, where could being disseminated through the region. In addition, molecular epidemiology determined by MLST revealed that hvKp UCO-494 belonged to the ST1161, which is apparently endemic to Chile since it has been detected previously in the country [43]. In the case of hmKv UCO-495, it was designated as ST173, which corresponds to a new ST that could be endemic to this geographical area. In consequence, further epidemiological studies are needed, in order to understand their prevalence and epidemiology in South America.

In the case of siderophore production, it has been demonstrated that yersiniabactin, salmochelin, and aerobactin are the most predominant in *K. pneumoniae* and *K. variicola* [44]. Specifically, the aerobactin system has four biosynthetic enzymes, *iucABCD*, and an outer membrane transporter, *iutA* [44]. Interestingly, epidemiological studies have shown a significant relationship between *iucABCD-iutA* with the hmKp phenotype; therefore, aerobactin is considered a substantive virulence factor in hvKp isolates [45]. However, the occurrence of multiple siderophore systems in hvKp strains suggests that siderophore systems in addition to Iuc-system play important roles in the pathogenesis of these microorganisms during either colonization or invasive processes [46].

Although all *Klebsiella pneumoniae* complex species could form mucoid colonies, it is well recognized the existence of two well-defined phenotypes. The classical (cKp/cKv) and hypermucoviscous (hmKp/Kv) phenotypes, both differentiated by their ability of forming a viscous and adhesive mucous string in solid media. Because of this, it is important to elucidate the mechanisms of CPS-production in hypermucoviscous *K. pneumoniae* strains that lack the *rmpA/rmpA2* genes and do not belong to the predominant K1 or K2 serotypes [47]. In this sense, Ernst *et al.* studied the impact of single-nucleotide polymorphisms of the *wzc* gene in the capsule biosynthesis, which could confer a hypercapsule production phenotype, enhancing virulence [16]; and additionally, contribute to the resistance to polycationic peptides, such as colistin [48]. On the other hand, diverse mechanisms are related with hypercapsule production, such as mutation in *wzc, rcsAB* and *lon* protease genes [49]. Our results showed a mutation in all of this gen in hvKp UCO-494. In this sense, some authors suggest that a single amino acid substitution in *wzc, rcsA* or *lon* protease genes could increase capsule production [16], and this mechanism could be related to the hypermucoviscous phenotype in *K. pneumoniae* UCO-494; however, this phenomenon has not been studied in *K. variicola.*

In the case of virulence, the *irp1* (polyketide synthetase) and *irp2* (iron acquisition yersiniabactin synthesis enzyme) encode for iron-repressible high molecular weight proteins that are involved in yersiniabactin production [4]. This siderophore system was first described for *Yersinia* species; however, they could be also present in other *Enterobacterales* [50]. It is believed that its dissemination occurred via horizontal gene transfer events since the responsible genes have been identified within pathogenicity islands, such as ICEKp, which is frequently identified in *K. pneumoniae* [2]. The mannose-sensitive type 1 fimbriae are common in *K. pneumoniae*. These fimbriae are encoded by *fim*-like genes, in which the major components are *fimA* and *fimH* that confer its ability to adhere to human mucosal or epithelial surfaces [51]. Furthermore, other important adhesin in *K. pneumoniae* is the mannose-resistant *Klebsiella*-like (type III) codified in the fimbriae cluster *mrkABCDFHIJ* [52]. This is considered as a virulence factor and contributor to mucous adherence, tissue colonization, and biofilm [53]. In our case, only UCO-494 *irp1* and *irp2* genes.

Importantly, biofilm-formation ability of hmKp contributes to hypervirulence, since hypervirulent strains generate more biofilms in comparison with less virulent isolates [54]. Specifically, biofilms provide protection against environmental conditions, such as desiccation, and also protect bacteria from the immune system action [46]. Accordingly, diverse studies associate biofilm phenotype to capsule, and/or fimbriae; however, it has been also demonstrated that the lack of capsule enhances biofilm-formation in *K. pneumoniae* [46]. Our results revealed that *K. pneumoniae* UCO-494 presented a low biofilm-formation ability, and at the same time displayed a lower *G. mellonella* killing ability in comparison to *K. variicola* UCO-495. Moreover, hvKp UCO-494 was susceptible to the serum activity, in contrary to hvKv UCO-495 that was resistant. However, hvKp UCO-494 produced more CPS in comparison with hvKv UCO-495, which is concordant with the bacterial-size capsule, in which hvKp UCO-494 has a thicker capsule than hvKv UCO-495. These discordant results suggest that more research is needed in order to establish the specific role of biofilm-formation and virulence in *Klebsiella* species. In this regard, some studies have demonstrated no significant differences in biofilm-formation ability between invasive (more virulent) and noninvasive (less virulent) *K. pneumoniae* isolates [55]. In another study, *K. pneumoniae* mutant strains with decreased biofilm production ability did not show any difference in their ability to survive serum activity, which reaffirms the need for further studies in this regard.

In conclusion, we identified the convergence of hypermucoviscous phenotype and MDR *K. pneumoniae* and *K. variicola* isolates in Chile. It is important to consider the relevance of these phenotypes since they are not normally screened by a routine laboratory. Moreover, our results demonstrate the relevance of *K. variicola* as pathogen, due to its antibiotic-resistance and virulence features. Moreover, our results suggest that the hypermucoviscous/hypervirulent phenotype of *K. pneumoniae*-complex isolates is the results of multiple mechanisms, including siderophores and biofilm-production, which have not been well elucidated yet. Our results remark the need for more detailed research of the mechanisms and epidemiology of hypervirulent strains, in order to elucidate the role of high-risk *K. pneumoniae*-complex lineages.
